# Long-Term Effects of Radiation on Lymphocytes and Risk of Opportunistic Infections

**DOI:** 10.7759/cureus.26887

**Published:** 2022-07-15

**Authors:** Gregory Swanson, Kendall Hammonds

**Affiliations:** 1 Cancer Center, Baylor Scott and White Health, Temple, USA; 2 Biostatistics, Baylor Scott and White Health, Temple, USA

**Keywords:** systemic toxicity, opportunistic infection, radiation therapy, immunosuppression, lymphopenia

## Abstract

Objectives

Lymphocytes are very sensitive to ionizing radiation. The long-term effects and the risk of permanent immune compromise are not well defined in spite of more than a century of therapeutic radiation. The contemporary analysis is made more difficult in that most patients also receive immunosuppressive chemotherapy.

Methods

Cohort-all patients that underwent a prostate biopsy from 2002 to 2007. Those (n=1118) with at least two blood counts, with one at a minimum of 10 years after biopsy, were included. We identified three groups: those that received no treatment (due to benign biopsy findings or active surveillance), those undergoing prostate cancer surgery, and those undergoing radiation therapy. Blood counts were collected and analyzed for differences with a median follow-up of 178 months. Also evaluated was the risk of opportunistic infections.

Results

The median granulocyte count gradually increased with time, with no difference between the groups. Overall, the median lymphocyte count decreased from baseline over time (with a slight rise at 20 years). For the no treatment and surgery groups, the lymphocyte counts declined, but due to the initial decline after radiation therapy, that group saw a slow improvement. By 20 years, there was no difference between the groups. Most patients’ counts remained in the normal range throughout. The risk of defined opportunistic infections was low (12%) with no difference between the groups.

Conclusion

Pelvic radiation has minimal lasting effects on lymphocyte and granulocyte counts. In addition, patients receiving radiation do not appear to be significantly immunocompromised.

## Introduction

Well recognized is that radiation suppresses lymphocytes [1]. Concerns about the long-term effects and risk of permanent immune compromise have existed for decades [2]. Although generally dismissed as unsubstantiated [3], the question has never been completely resolved and consists of two parts-does radiation therapy suppress lymphocytes long-term, and are those patients immunocompromised? Most data are contaminated by the inclusion of chemotherapy, which itself has known immunosuppressive effects. There is limited data on the long-term effects of radiation. We evaluated the long-term effects of pelvic radiation therapy on lymphocyte count and immunity.

## Materials and methods

With approval from the institutional review board, our database consisted of all patients that underwent a prostate biopsy at Baylor Scott and White in Central Texas from 2002 to 2007, with the goal of having at least 10 years of follow-up. We included patients with both benign biopsies and those with the finding of prostate cancer. For those with cancer, treatment consisted of active surveillance, radical prostatectomy, cryotherapy, and radiation therapy. Initial analysis (data not shown) showed patients treated with cryosurgery (n=36) had similar results to those treated with surgery, so those groups were combined. Androgen deprivation was used non-consistently and, having previously shown there was no significant effect on blood counts [4], those patients were included in the active surveillance/no cancer group. Patient characteristics are shown in Table 1. We collected baseline and subsequent blood counts. The normal range in our lab for granulocytes is 1.92-8.64 × 10^9^/L and for lymphocytes is 0.72-4.32 × 10^9^/L. Pre-biopsy counts were recorded if available, but the minimum criteria were for a baseline complete blood count before treatment or within the first five years from biopsy for untreated patients, followed by two subsequent blood counts, with the final one at least 10 years after the biopsy or treatment. To account for other disease processes that might affect the blood counts, the medical records for each patient were screened. Every hematology/oncology, rheumatology, infectious disease, urology, and transplant surgery note was reviewed. Excluded were all chemotherapy and immunotherapy patients, whatever the underlying cause (such as cancer, Crohn’s or ulcerative colitis, and rheumatoid arthritis). For practical reasons (the number of blood counts available), we were seeking overall trends and, if available, recorded at least one count every two years. Excluded were labs with a WBC >15 because of the obvious infection implication. We excluded brachytherapy patients because their effects on counts are uncertain.

For the included patients, we searched the billing records for International Classification of Diseases (ICD)-9 and ICD-10 codes (to capture pre- and post-October 1, 2015 data, respectively) for opportunistic infections that occur in the immunocompromised (Table 1), as classified by the United States Center for Disease Control (CDC) [5]. In addition, we included the “opportunistic malignancies” of Kaposi sarcoma and non-Hodgkin’s lymphoma (including CNS lymphoma) [6]. The codes used are not completely specific to the diseases listed in Table 1, so for each specific billing date, the medical records were individually searched to determine the exact diagnosis. Specially, in the review for pneumonia, the criteria for an opportunistic infection are the finding of mycobacterium, pneumocystis, histoplasmosis, and coccidioidomycosis (which actually have separate codes) or two bacterial episodes in a single year. For Candida infections, the criteria are specific for oral/esophageal but not inclusive of skin. For infectious diarrhea, it is for specific organisms such as Shigella, Salmonella, and Clostridium difficile.

**Table 1:** Center disease control for opportunistic infections, ICD-9 and 10 codes searched. ICD: International Classification of Diseases.

Disease	ICD-10	ICD-9
Infectious diarrhea	A09	0090-0093
Bartonellosis	A449	0880
Candidiasis	B370-379	1120-1129
Chaga’s disease	B570-575	0860-0861
Pneumonia	J120-129; J150-159; J180-189	4800-4809
Cryptococcosis	B450-459	1175, 3210
Cryptosporidiosis	1072	0074
Cystoisosporiasis (isosporiasis)	A073	0072
Coccidioidomycosis	B380-389	1140-1149
Cytomegalovirus	B250, B259	4841, 0785
Hepatitis B, hepatitis C	B180-189	07023-07059
Herpes simplex	B000-009	0540-0549
Histoplasmosis	B392-399	11505-11599
Herpesvirus 8	B1001-1089	05821-05889
Papillomavirus (+ others)	B970-9789	0790-07999
Leishmaniasis	B550-559	0850-0859
Malaria	B530-538; B520-529	0843-0847; 0842-0849
Microsporidiosis	B600-608	1368
Mycobacterium avium	A310-A319	0310-0319
Mycobacterium tuberculosis	A150, A155	01100-01196
Pneumocystis pneumonia	B59	1363
Progressive multifocal leukoencephalopathy	A812	0463
Syphilis	A510-A515	0910-0929
Talaromycosis (penicilliosis)	B359	1109
Varicella-zoster	B010-B019	0520-0529
Toxoplasma gondii	B580-B589	1300-1309
Kaposi sarcoma	C460-469	1760-1769
Non-Hodgkin’s lymphoma (includes CNS lymphoma)	C8290-8298; C8300-8339; C8350-8359; C8370-8389; C8460-8479; C8400-C8418; C8580-C8588; C969; C96Z	20000-20088; 20200-20298

## Results

One thousand one hundred eighteen patients met the criteria for inclusion. The mean age of all the patients' undergoing biopsy was 66.7 years (range 38.7-86.7). Fifty-two percent (n=580) were diagnosed with prostate cancer and 48% (n=538) without. Fifty-four percent (n=605) did not have cancer or received no treatment (active surveillance), 25% (n=314) underwent surgery (278 prostatectomy, 36 cryosurgery), and 18% (n=199) received radiation therapy. The median follow-up was 178.0 months (14.8 years).

Overall, the median granulocyte count gradually increased with time, while the median lymphocyte count decreased with time, except for long-term (20-year) survivors that had a slight rise (Table 2).

**Table 2:** Overall change in median counts.

	Granulocyte count (× 10^9^/L)			Lymphocyte count (× 10^9^/L)		
		Range	Change		Range	Change
Baseline	3.88	0.76-12.8		1.79	0.35-4.37	
5 years	3.94	0.99-10.9	0.63%	1.68	0.44-5.46	−3.39%
10 years	4.06	0.46-10.89	1.28%	1.68	0.35-5.68	−5.06%
15 years	4.29	0.92-11.30	6.66%	1.64	0.46-4.16	−7.12%
20 years	4.19	1.34-10.78	6.13%	1.63	0.44-4.17	1.63%

We next considered the context of deviation from normal. Our normal laboratory range for granulocytes is 1.92-8.64 × 10^9^/L and lymphocytes is 0.72-4.32 × 10^9^/L. In this context, 2.3% had granulocytes below normal at baseline and 0.7% had lymphocyte counts below normal. For granulocytes, 1.8-2.0% remained below normal through 15 years, with 0.6% below normal at 20 years. Overall, for lymphocytes, the percent below normal did not change at five years and only slightly (1.5-2.2% were below normal) at 10 through 20 years. For granulocytes, 1.8% were below normal at five years, and 2% were below normal at five and 10 years.

For changes in blood counts for each of the three groups (no treatment, surgery, and radiation therapy; Table 3), for granulocytes, there was a gradual increase in counts over time. Between the groups, there was no difference in counts at baseline out to 20 years, except for some minor differences at five years (p=0.0372). Also, in all three groups, there was a gradual decline over time in lymphocyte counts for the no treatment and surgery groups, but the radiation therapy group improved with time. Due to the larger initial decrease, lymphocyte counts in the radiation patients were significantly lower initially and out to 10 years, but with the gradual improvement, they were not significantly different later on.

**Table 3:** Granulocyte and lymphocyte count with time for no treatment, surgery, and radiation therapy. *Median change from baseline (Kruskal-Wallis test).

	Granulocytes (median) (× 10^9^/L)								
	Baseline	Five year	Change*	10 year	Change*	15 year	Change*	20 year	Change*
No treatment	3.91	4.00	1.40%	4.03	0.83%	4.29	6.00%	4.16	2.36%
Prostatectomy	3.82	3.71	−2.50%	3.98	0.49%	4.20	7.64%	4.27	8.75%
Radiation therapy	4.05	4.08	1.28%	4.21	5.68%	4.61	8.26%	4.32	7.22%
P-value	0.3442	0.0372	0.6416	0.1401	0.5071	0.10601	0.03553	0.9243	0.7614
	Lymphocytes (median) (× 10^9^/L)								
No treatment	1.8	1.77	−1.88%	1.72	−4.07%	1.68	−6.29%	1.67	−11.28%
Prostatectomy	1.77	1.71	−1.27%	1.73	−2.44%	1.60	−8.11%	1.63	−3.96%
Radiation therapy	1.77	1.41	−18.42%	1.46	−17.60%	1.44	−13.08%	1.39	−19.66%
P-value	0.6933	<0.0001	<0.0001	<0.0001	0.0009	0.0060	0.1421	0.3055	0.0925

A broader consideration is whether patients' counts were in the normal range. The implication is that blood counts in the normal range are adequate for health. For granulocytes, <5% of the patients in each group had counts less than the normal range with no significant differences between the groups except for a significant difference at 15 years that disappeared at 20 years.

Although there was a general decline in lymphocyte count from baseline, most patients (>95%) remained in the normal range with no significant difference between the groups throughout the duration of follow-up.

In the detection of opportunistic infections, on the initial screening, 326 (29%) patients had a positive screened billing code, with no difference between the three groups (p=0.9304). Thirty-seven patients had two codes and one had three, for a total of 364 code incidents. Most incidents were for pneumonia (61%), with 12% for candida, 10% for herpes, and 9% for infectious diarrhea. After screening each of these occurrences by review of the records, 114 patients met the strict criteria (CDC) for an opportunistic infection, which was not different between the three groups (p=0.0882). Seven patients were coded for two diseases. The most common coded diseases were herpes (31%), oral-esophageal candida (26%), and recurrent pneumonia (17%) (Table 4).

**Table 4:** Occurrence of opportunistic infections in each cohort.

	No treatment	Surgery	Radiation	Total
Total patients	605	314	199	1118
Herpes	18	11	6	35
Oral/esophageal candida	20	2	8	30
Recurrent pneumonia	10	4	3	17
Infectious diarrhea	10	1	0	11
Hepatitis	8	0	2	10
Lymphoma	2	2	3	7
Tuberculosis	2	2	2	6
Kaposi sarcoma	1	0	0	1
Cytomegalovirus	0	1	0	1
Coccidioidomycosis	1	0	0	1
Histoplasmosis	1	0	0	1
Strongyloidiasis	1	0	0	1
Total	74 (12%)	23 (7%)	24 (12%)	121 (11%)

## Discussion

While it has been long established that radiation treatment causes a decline in lymphocyte numbers [7], the long-term effects have not been well defined. With the rapid escalation of chemotherapy use in the 1980s, including patients treated with radiation therapy, out of necessity, almost all the studies evaluating lymphocyte recovery after radiation alone predate the mid-1980s. Most are small studies with only modest follow-ups. One of the earlier studies [8] evaluated 56 patients that received post-mastectomy radiation. When measured two or more years later, there was still a significant (p>0.01) lymphocyte decline from baseline (−37% and −43% for patients without and with recurrence, respectively). In a later study of breast cancer patients with radiotherapy [9], eight had baseline levels and while they had a decline at three years or later (−26%), it was non-significant. Then, in a mixed population of patients (breast, prostate, cervix, testes cancers) [10], 14/15 patients measured within three years of treatment had depressed lymphocyte counts (more than two standard deviations below normal), but after three years, only 33% (6/9) were below normal. We had previously evaluated the short-term effects on lymphocytes [1]. While median lymphocyte count decreased by 65% at the end of treatment, by three months, it had already started to improve (median −50% below baseline). Based on normal ranges, 60% of the patients were below normal at the end of treatment, which improved to 28% below normal at three months. With longer follow-up (median 28 months), it had incrementally improved to 14% below the normal range.

In the current study with a much longer follow-up (median 178 months), all three groups experienced lymphocyte counts below their initial baseline. The trend was greater in those that had received radiation, but it was not significantly different from the non-radiation patients at 15 and 20 years (Table 3). In addition, 96% of the surviving radiation patients’ lymphocyte counts were in the normal range at 15 years and 100% at 20 years, which was not significantly different than those that did not receive radiation (Table 5). We could find no comparative data in the literature.

**Table 5:** Patients in normal range over time.

	Baseline	Five years	10 years	15 years	20 years
Granulocytes					
No treatment	96.18%	96.79%	96.79%	96.19%	95.76%
Prostatectomy	93.32%	97.01%	95.06%	96.37%	100%
Radiation therapy	97.78%	97.67%	96.25%	87.23%	100%
P value	0.2561	0.90.33	0.1525	0.0118	0.5870
Lymphocytes					
No treatment	99.24%	99.79%	98.44%	98.70%	96.72%
Prostatectomy	98.86%	97.96%	99.21%	98.67%	100%
Radiation therapy	100%	98.86%	95.12%	95.83%	100%
P value	0.6751	0.0582	0.0.0814	0.2766	0.3872

Similarly, there is no comparable long-term data on granulocytes and neutrophils. In our study, the granulocyte count gradually increased from baseline in all the patients, with no significant difference between patients that did or did not receive radiation. There was also no difference in those below normal range between the groups throughout, except at 15 years when the radiation patients had declined by about 10%, which was a significant difference. This had resolved at 20 years.

While we have shown that the long-term effects on lymphocytes and granulocytes are marginal, the remaining question is whether there are lasting effects on immunity. The measurement of immunity is complex, but there is some consensus on infections and cancers that occur in patients that are considered immune-compromised. Based on current definitions of opportunistic infections in the immune-compromised (CDC), we searched our records for occurrences. Overall, 11% of the patients had occurrences that met the definition (Table 4), but there was no significant difference as to whether the patients had radiation or not. The patient numbers clearly are not large enough to detect subtle differences, but it is reassuring. There are no other studies with large patient numbers that address this.

As with all retrospective studies, there are pitfalls. The obtaining of blood counts over the years was sporadic, some were collected routinely, others because there were health concerns. Some patients had a few labs, others had many. Some had none at all. With longer follow-up, surviving patients were obviously the most healthy and their blood counts would be expected to reflect that. Given that some of these biases would persist even in a prospective study, the best recourse would be large-volume population studies. As it is, we have completed the most complete and comprehensive evaluation to date, so it represents a good baseline.

## Conclusions

We have seen that lymphocyte counts gradually decrease in all patients as they age. The change is greatest with radiation but mostly within the normal laboratory range. As a result, there is no significant difference between those that did and did not have radiation. Immunocompromised patients are known to have an increase in opportunistic infections. We could detect no long-term difference in opportunistic infection between those that did and did not receive radiation. We have shown that pelvic radiation has minimal lasting effects on lymphocyte and granulocyte counts. In addition, patients receiving radiation do not appear to be significantly immunocompromised.
